# Differential metabolomic effects of progestagen-based estrus synchronisation in South African Dohne Merino ewes

**DOI:** 10.1007/s11250-026-05198-0

**Published:** 2026-07-10

**Authors:** R. Shingange, C. Visser, M. Wooding, E. C. Webb

**Affiliations:** 1 Department of Animal Science, University of Pretoria, Pretoria, South Africa; 2 Department of Chemistry, University of Pretoria, Pretoria, South Africa

**Keywords:** Assisted reproductive technology, Intravaginal pessary, Dysbiosis, Cervicovaginal fluid, Metabolomics

## Abstract

This study represents one of the first to comprehensively profile the homeostatic cervicovaginal metabolome of South African Dohne Merino ewes using liquid chromatography high resolution mass spectrophotometry (LC-HRMS) and compare that metabolome immediately after estrus synchronisation using progestagen intravaginal pessaries. As the use of assisted reproductive technologies gains traction in various livestock production systems, characterising their effects using novel omics approaches is crucial to physiology, welfare and production. An ewe has well-developed metabolomic cascades that are homeorhetically responsive to the stress caused by assisted reproductive treatments in order to maintain homeostasis. The cervicovaginal fluid of sixty ewes was sampled by transvaginal FLOQSwabs before pessary insertion as well as immediately post-pessary removal. These swabs, along with field, solvent and swab blanks, underwent extraction in acetonitrile and drying under nitrogen prior to reconstitution. Using one-way ANOVA with Tukey’s HSD post-hoc analysis and PERMANOVA, this study found no significant metabolomic features prior to treatment (*P* > 0.05). However, after pessary removal, 32 features were reported to be different between the CIDR-, sponge-treated and control group (*P* < 0.005 and False Discovery Rate < 0.05). Features were identified at four levels including at verification with pure chemical standards. Progesterone was found to be highest in the CIDR-treated group (*P* < 5.0 × 10^− 48^) while linolenic acid, an immune modulator, was highest in the sponge-treated group (*P* < 5.0 × 10^− 3^). These findings present a novel understanding of the metabolomic fluctuations that occur within the cervicovaginal microenvironment in response to progestagen synchronisation protocols, which are a routine part of management in the production systems of sheep and various other species of livestock. These stark metabolomic differences highlight endocrine and immune changes that are triggered by the CIDR or sponge and warrant further characterisation of the physiological and welfare implications of these technologies.

**Supplementary Information:**

The online version contains supplementary material available at 10.1007/s11250-026-05198-0.

## Introduction

In livestock research, only 10% of studies using metabolomic analysis have aimed to characterise healthy animals and identify homeostatic metabolites in different bodily fluids or tissues (Goldansaz et al. 2017). The current evidence base thus provides limited guidance for the differential categorisation of a metabolome as dysbiotic, characterised by the decreased production of beneficial metabolites and an increase in harmful or inflammatory metabolites (Pyles et al. 2021).

The metabolome encompasses all metabolites that are present within a biological system and provides insights into that animal’s physiological state. In the context of the cervicovaginal fluid (CVF) of ewes, the metabolites that are present in the reproductive tract have the potential to influence reproductive outcomes significantly as different variables in the ewe’s reproductive tract will naturally fluctuate as she attains puberty, mates, conceives and undergoes parturition (Esposito et al. 2014; Zhai et al. 2023). However, the metabolome of an ewe that has, for example, endometritis, will have a CVF metabolome in which specific metabolites associated with the condition are detectable even prior to the manifestation of clinical signs of the pathology, which is important for the development of interventions targeting related biosynthetic pathways (Ioannidi et al. 2020; Hao 2024).

The variety of biosynthetic pathways that exist within an organism have one goal: To manifest a phenotype that best supports homeostasis. These pathways use genetic transcription to produce necessary proteins and other compounds that participate in metabolism (Fontanesi 2016). Metabolites are necessary for an animal to display an observable set of characteristics ranging from increased immune activity in the aim to mitigate infection to reduced digestive activity to mitigate heat production (Kim et al. 2024). Thus, metabolism is a unique pathway that traverses the up- or down-regulation of genes until phenotypic expression, which will necessarily reflect the state of the physiological environment (Fontanesi 2016; Goldansaz et al. 2017).

Intravaginal pessaries which remain in the vaginal canal for a 5 to 14 day period are routine parts of estrus synchronisation protocols, aimed at triggering treated female livestock to display willingness to mate and ovulate at a predictable time (Echternkamp and Thallman 2011; Fernandez-Novo et al. 2021). Intravaginal pessaries for estrus synchronisation are either Controlled Internal Drug Release devices (CIDRs), made of silicon, or sponges, made of polyurethane (Tschopp et al. 2022). These foreign objects inhabit an otherwise undisturbed microenvironment for an extended period and their physiological and physical stimuli will trigger a response in the ewe aimed at mitigating the shift from homeostasis (Esposito et al. 2014; Bovo et al. 2025).

The aims of this study were thus to firstly characterise the homeostatic CVF metabolome in a flock of Dohne Merino ewes that had not previously been subjected to intravaginal pessary estrus synchronisation and were of varying parities. Secondly, this study aimed to compare the metabolome of the ewes’ CVF at pessary removal when treated with either a CIDR or sponge intra-vaginal pessary, where injection-based estrus synchronisation served as a control treatment. This comparison was made between treatment groups as well as between sampling points.

## Materials and methods

### Ethical approval

Ethical approval was obtained from the University of Pretoria’s Animal Ethics Committee (NAS 162/2022) as well as a Section 20 permit from the South African Department of Agriculture, Land Reform and Rural Development (12/11/1 2777KL).

### Study area

This study was conducted at the University of Pretoria’s Innovation Africa farm in South Africa from June to December 2024.

### Animals and their management

Ewes (*n* = 60) were randomly selected from a pure bred Dohne Merino flock. Following collection of baseline animal characteristics, animals were allocated to treatment groups using stratified randomisation. The ewes’ average age was 3 years old. The CIDR-based treatment group had a mean weight of 55.24 kg; the Sponge-based treatment group, 55.5 kg; and the non-pessary treatment group which was considered the control group, 54.79 kg. All groups had 30% nulliparous ewes, 10% primiparous, and 60% biparous to pentaparous ewes. The non-nulliparous ewes had an average of 82.96 days since their most recent partition, sufficient time for the reversion of metabolic state and uterine histology to pre-partum and pre-gestational conditions (Pesántez-Pacheco et al. 2019; Evkuran Dal et al. 2020). Treatment groups were housed in the same pen and grazing-group over the period of the study, with ad-lib access to water and grazing on kikuyu (*Cenchrus clandestinus*) grass.

### Estrus synchronisation

The estrus synchronisation protocol used is tabled in Table 1. For CIDR-based synchronisation, a CIDR (EAZI-BREED™ CIDR^®^ Sheep Insert, Zoetis) was used; for sponge-based synchronisation, a sponge (GenoCo, South Africa) was used. For the prostaglandin-based control estrus synchronisation, Estrumate (Estrumate ^®^, Merck Animal Health) was administered to regress the corpus luteum if present.

All ewes received 15 mL Ovi-Thrive (Ovi-Thrive Sheep and Goats with Copper, Ashkan Consulting (Pty) Ltd) prior to treatment commencement as well as melatonin, to mitigate possible differences in response caused by insufficient activity of the HPG-axis due to photoperiod or nutritional deficits (Cevik et al. 2017; Berean et al. 2021). At pessary removal, all treatment groups received Chronogest (Chronogest ^®^ PMSG 6000 IU, Merck Animal Health), to stimulate the ovulation of a dominant follicle.

**Table 1:** Experimental design and estrus synchronisation treatment groups

Treatment group	CIDR-treated	Sponge-treated	Control
Sample size	20	20	20
Day 0	15 mL OviThrive (oral) and 1 mL / 50 kg melatonin (subcutaneous)		
Day 16 (*Prior to treatment CVF metabolome sampling*)	CIDR insertion	Sponge insertion	2 mL Estrumate (intramuscular)
Day 24 (*Immediately post-treatment CVF metabolome sampling*)	CIDR removal and 2.5 mL Chronogest (intramuscular)	Sponge removal and 2.5 mL Chronogest (intramuscular)	2 mL Estrumate (intramuscular) and 2.5 mL Chronogest (intramuscular)

### Cervicovaginal metabolome

Before the treatments listed in Table 1 were administered, CVF samples were collected from all ewes (*n* = 60), regardless of treatment group. These samples were considered as homeostatic metabolomes. For all sampling, an unused sterile, packaged speculum was used for each ewe, where an 80 mm FLOQSwab (Copan, Brescia, Italy) was used for transvaginal sampling before removal of the speculum. While securely restrained, the ewe’s vulva was parted and the speculum inserted into the posterior vaginal canal. A FLOQSwab was then inserted intravaginally and through the *external os* of the cervix, as visualised via the speculum. Discomfort was minimised for the ewe by placing her in sturdy restraints while standing. The swab insertion was along the long axis of the vaginal canal. Once placed at the posterior cervix, the swab was rotated along its horizontal axis as well as along the circumference of the cervix, with equal rotations clockwise and counterclockwise to allow the swab’s fibres to make full contact with the cervical walls. This was achieved using gentle but consistent lateral pressure. The swab was then retracted until exiting the cervix, and the rotational swabbing movement repeated in the *vagina proper*. This rotational movement increased contact with cervical and vaginal tissue, ensuring absorption of the biological material that was present. The swab was carefully withdrawn in a smooth, controlled motion to avoid contamination from contact with the speculum, vestibule or vulva, and placed in a sterile dry tube for transportation.

#### Reagents and chemicals

A steroid internal standard mix was purchased from Microsep (Waters™, Microsep, South Africa) as the pure chemical standard. The standard was prepared as per manufacturer specifications and injected with the lowest target analyte concentration being 2 ng/mL and the highest 3000 ng/mL. All solvents used, including acetonitrile (ACN), methanol (MeOH), and water, were sourced from Romil (Romil-UpS™, Microsep, South Africa).

#### Metabolite extraction

**Fig. 1:**
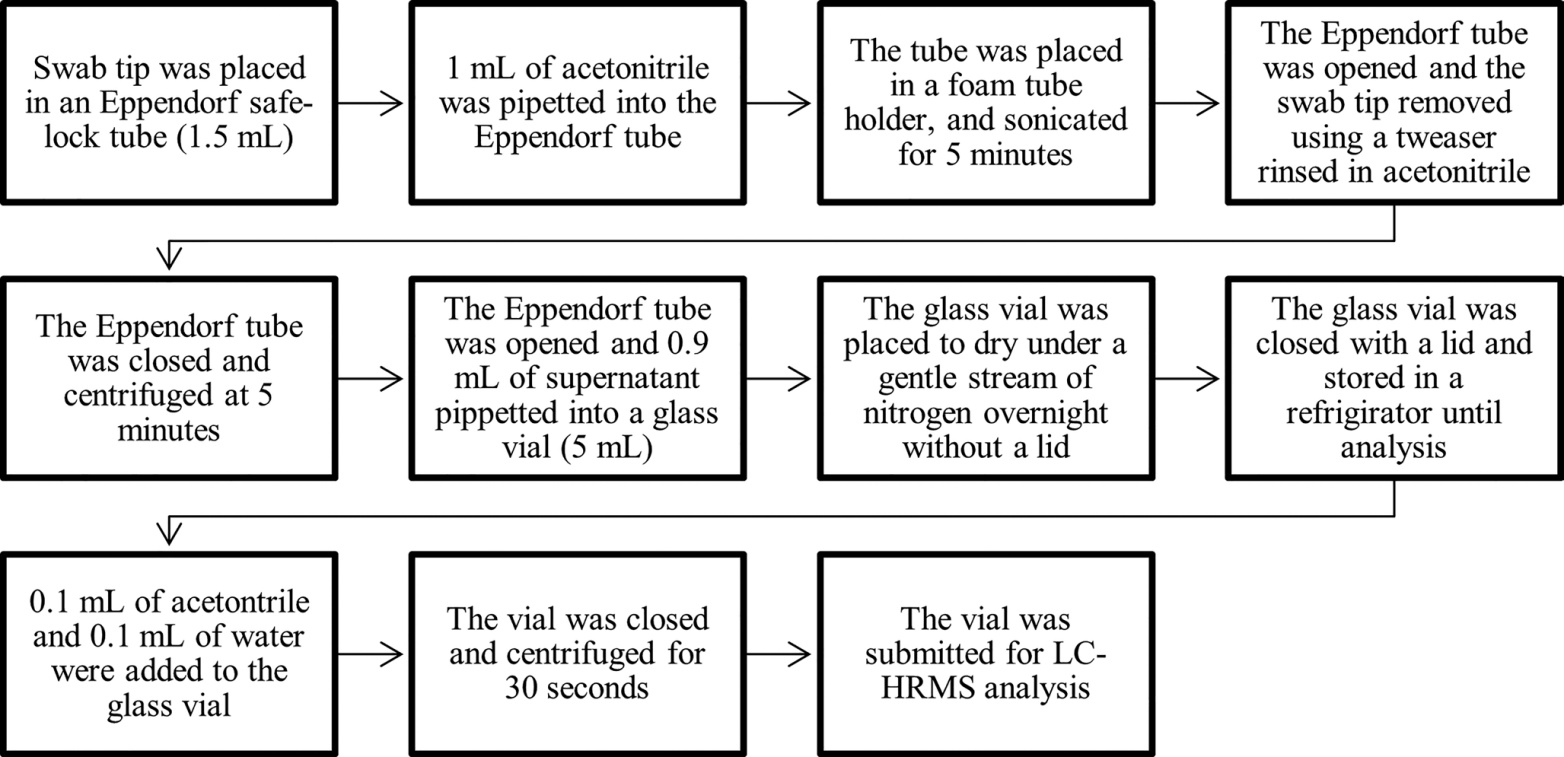
Metabolomic extraction and reconstitution of swabs. Resultant multivariate feature tables were created using the UNIFI Scientific Information System (UNIFI) (Waters Inc. 2025) for further statistical processing

After sampling, swabs were processed as per the diagram below (Fig. 1).

#### Feature identification

Features were identified across four levels (Salek et al. 2013): Level 1 is a feature for which a pure analytical standard was compared using brand specific methodology in the laboratory that was also used to process biological samples using UNIFI and MassLynx Software (Waters Inc. 2025). Level 2 is a feature annotated using accurate mass, fragmentation patterns, and MS libraries wherein the tolerated mass difference was < 5 ppm and calculated as:

$$\:\triangle m=mz\:\times\:\:\frac{ppm}{{10}^{6}}$$

The mass to charge ratio (m/z) and retention times (rt) (minutes) of detected features were obtained from UNIFI and consolidated using its multivariate marker option with a m/z tolerance of 5 ppm and rt of 0.02 min. These features were normalised by quantile (Sun and Xia 2024) without filtering in MetaboAnalyst’s (version 6.0) R Studio (version 2025.09.2 + 418) package (Pang et al. 2024; Posit Team 2025). A one-way ANOVA was used to perform significance testing for each sampling point, wherein raw p-values between groups per feature were generated in R Studio (Ritchie et al. 2015; Posit Team 2025). The feature’s m/z, rt, and p-value were used to generate a peak list table to be used in MetaboAnalyst’s Functional Analysis module in R Studio, as this module supports compound identification for untargeted metabolomics using the mummichog algorithm (Pang et al. 2024; Posit Team 2025). The full peak list was uploaded, as opposed to only significant features (*P* < 0.05), for the model to identify plausible metabolite sets / pathways as determined by differences at compound level (Li et al. 2013). Further, level 3 is a feature only annotated using chemical formula as determined by spectral data in UNIFI including the iFit Confidence (%) to indicate consistency of the feature’s m/z and rt with that expected of the chemical formula giving a putative compound class assignment (Broadhurst et al. 2018). Level 4 is a feature for which no other information apart from m/z and rt were obtained.

#### Quality control of samples and feature filtering

All cervicovaginal samples were duplicated by the successive use of two swabs per ewe both prior to treatment at Day 16 and immediately post treatment at Day 24 to minimise experimental error plausibly correlated to a specific swab. Additionally, to detect meaningful differences in biological samples and detect both enriched and depleted metabolites while removing ambiguous or noise-level signals, field blanks, solvent blanks and swab blanks were included in analysis (Zheng et al. 2019). The field blank was taken by removing the sterile swab from its sterile holder, and gently waving the swab near the ewes and along the longitude of the restraining pen to determine if the observed results reflect actual differences between treatment groups or if they are influenced by environmental contamination at each sampling point (Hu et al. 2014). Additionally, features of the solvent blank were determined by running the solvents used for analysis through the same sample processing procedure used for sample swabs, without any sample present. This allowed the detection of impurities or contaminants that may have been introduced by solvents or reagents (Martínez-Sena et al. 2019). Apparatus may have also been a cause of background noise and contamination thus swab blank features were obtained by processing a sterile swab in the laboratory as a biological sample.

To filter metabolites, the group and blank detection rate; the ratio between a feature’s observed mean across non-blank samples and in blank samples; the difference between a feature’s observed mean across non-blank samples and in blank samples; and a feature’s mean intensity across non-blank samples were used. This filtering methodology was adapted from Schiffman et al.’s data adaptive filtering procedures for untargeted LC-HRMS data (Schiffman et al. 2019) as detailed in Table 2. A feature must have met all criteria for either the high- or low-quality category to be classified as such.

**Table 2:** High- and low-quality feature criteria for data adaptive blank sample filtering

Criteria					Reference
Variable	Definition	Rationale	High-quality feature	Low-quality feature	
Group detection rate	The proportion of non-blank samples of a treatment group that a feature is found in.	A feature was classified as high-quality if it was present in 80% of samples per treatment group and as low-quality if it was detected in less than 30% of samples per treatment group.	> 0.8	< 0.3	(Schiffman et al. 2019; Liu et al. 2020)
Blank detection rate	The proportion of blank samples in which that feature was present.	A feature will be low quality if it is present in more than half of the blank samples, implying it is a background contaminant.		> 0.5	(Schiffman et al. 2019)
A feature’s observed mean in non-blanks : That feature’s observed mean in blanks	The ratio between a feature’s observed mean across non-blank samples and in blank samples	To ensure low quality features are those not enriched in non-blank samples and are thus those that do not distinguish blank samples from cervicovaginal samples.	> 2	< 0.5	(Mahieu and Patti 2017; Liu et al. 2020)
(A feature’s mean in non-blank samples) – (that feature’s mean in blank samples)	The difference between a feature’s mean across non-blank samples and that feature’s mean in blanks	To ensure that a high-quality feature is enriched in non-blank samples compared to blanks.	> 1	< 0.5	(Liu et al. 2020)
A feature’s mean intensity in non-blank samples	The abundance of a feature across non-blank samples	To detect features that have low abundance or those that have inconsistently integrated peaks		< 1	(Martínez-Sena et al. 2019)

### Statistical analysis

Statistical significance was reported using exact and threshold p-values, with significance declared at *P* < 0.05. The MetaboAnalyst (version 6.0) R Studio (version 2025.09.2 + 418) package was used to perform multivariate linear regression and obtain p-values and adjusted p-values (P). Features were considered significant within samples if *P* < 0.05 and False Discovery Rate < 0.05. Two-dimensional (2D) Score Plots were generated using MetaboAnalyst (version 6.0) to visualise multivariate patterns in the dataset (Pang et al. 2024), with group differences assessed using a Permutational Multivariate Analysis of Variance (PERMANOVA) in R Studio (Posit Team 2025). Where features were significant between treatment groups and between sampling points (*P* < 0.05), Tukey’s HSD post-hoc analysis was done in R Studio. Tukey’s HSD values were reported as post-hoc significance figures and not as measures of variability while other measures of variability were reported as standard deviation (SD). Pearson correlation heatmaps for features obtained prior to and immediately post-treatment was generated using MetaboAnalyst (version 6.0) (Pang et al. 2024).

## Results

Prior to treatment, there was a notable lack of significant differences in the CVF metabolome of ewes between and within the CIDR-, sponge-treated and control groups (*P* > 0.05). Comparatively, 455 significant features were detected immediately post-pessary removal (*P* < 0.05) and after filtering, 32 features were reported (*P* < 0.005 and FDR < 0.05).

**Fig. 2:**
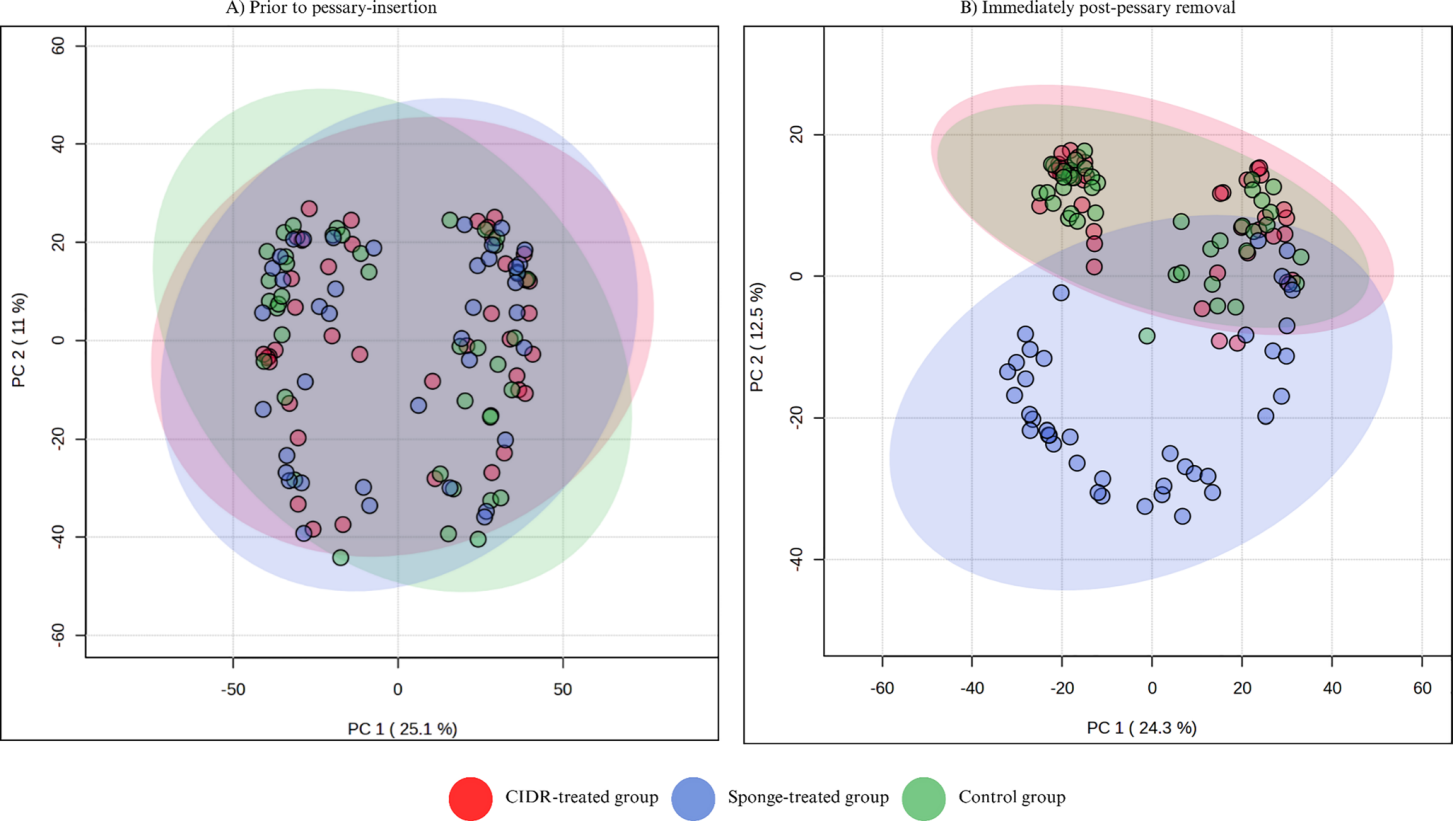
2D Score Plot of the observed features in the cervicovaginal metabolomic of the control, sponge-treated and CIDR-treated Dohne Merino ewes prior to pessary insertion (**A**) and immediately post-pessary removal (**B**)

Prior to pessary insertion, Fig. 2(A) showed heavy overlap between samples from the three treatment groups. The coloured ellipses also indicated no clear separation between treatment groups based on the principal components having no explicit outliers. However, immediately post-pessary removal, distinct differences between treatment groups emerged in Fig. 2(B). Samples of the control and CIDR-treated groups clustered together with notable overlap while samples of the sponge-treated group formed a distinct cluster mainly separated along PC2, highlighting that the sponge-treated group had a different metabolomic profile from the CIDR-treated and control groups.

Additionally, Table 3 highlights statistically significant between-group differences as per PERMANOVA. Prior to pessary-insertion, there was no significant difference between different treatment groups (*P* > 0.05). This is also evidenced by similar F-values, indicating low group separation, as well as low R^2^ values, indicating weak group structure.

Conversely, notable differences between treatment groups are seen immediately post-pessary removal. The CIDR-treated group had a metabolomic profile similar to that of the control group (*P* > 0.05) as evidenced by the very low effect size (R^2^ = 0.003) as well as the low F-Value (F-Value = 0.201). This is in contrast to the significant differences seen between the CIDR-treated group and the sponge-treated group, as well as between the control and sponge-treated group (*P* < 0.005) where high F-Values indicated clear group separation (F-Value = 26.945 and 26.353, respectively) and a moderate effect size indicated meaningful contribution of the treatment to the group’s metabolome (R^2^ = 0.256 and 0.253, respectively). The significant adjusted p-value further emphasises the statistical robustness of the differences between the sponge-treated group and the CIDR-treated and control group (*P* < 0.005).

**Table 3:** The PERMANOVA analysis of the effect of CIDR and sponge estrus synchronisation treatments compared to a control group in Dohne Merino ewes prior to pessary insertion and immediately post-pessary removal

Group comparison	Prior to pessary-insertion				Immediately post-pessary removal			
	F-Value	*R* ^2^	*P*	Adjusted *P*-value	F-Value	*R* ^2^	*P*	Adjusted *P*-value
CIDR-treated vs. Control	0.152	0.002	0.851	0.947	0.201	0.003	0.701	0.701
CIDR-treated vs. Sponge-treated	0.056	0.001	0.947	0.947	26.945	0.256	*0.001	0.002
Control vs. Sponge-treated	0.077	0.001	0.936	0.947	26.353	0.253	*0.001	0.002

Key:

*P < 0.005

F-statistic (F-Value)

Effect size (R^2^)

**Fig. 3:**
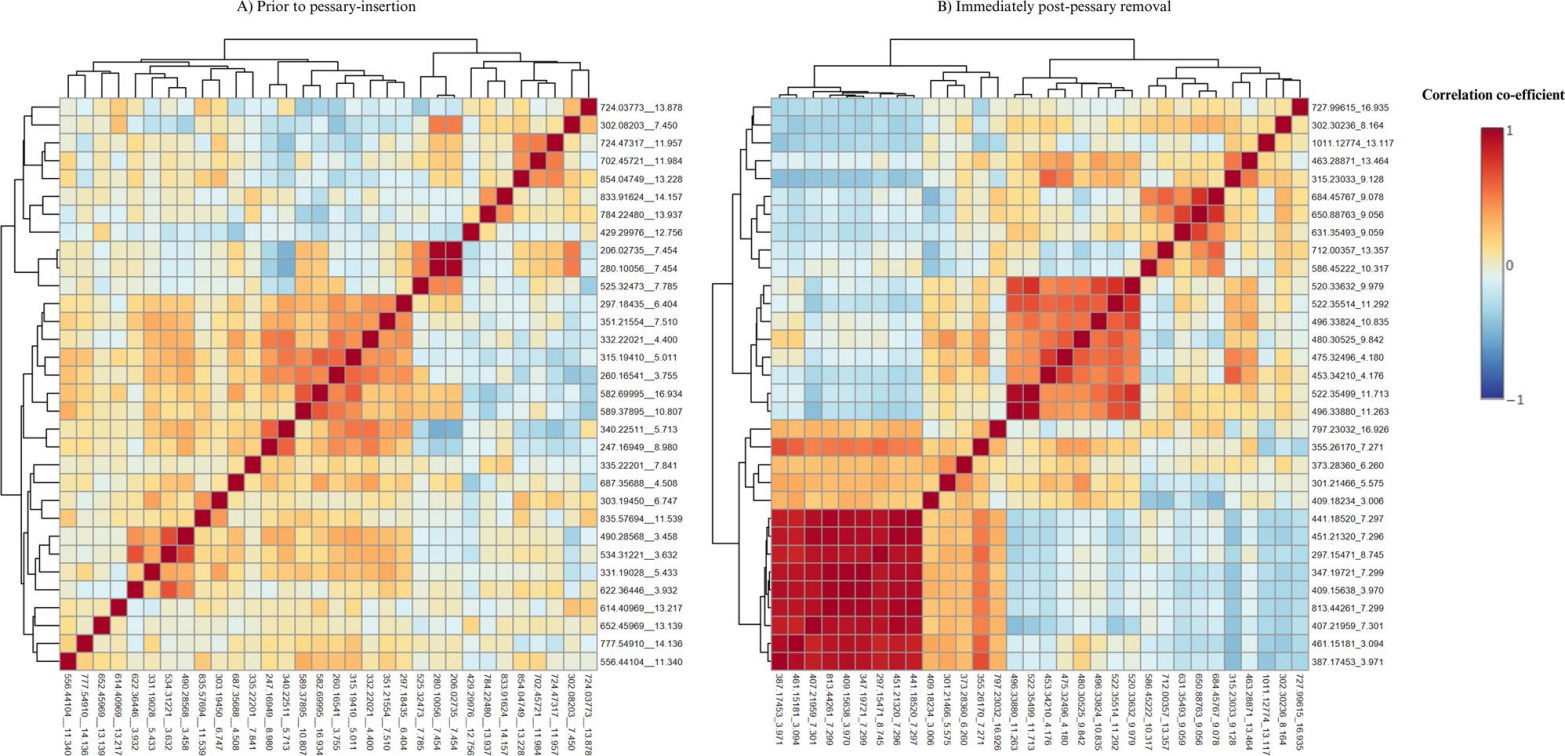
Correlation heatmaps of the observed features in the cervicovaginal metabolome of the control, sponge- and CIDR-treated Dohne Merino ewes prior to pessary insertion (**A**) and immediately post-pessary removal (**B**)

Comparative inspection of two correlation heat maps in Fig. 3 revealed differences in structure of the metabolomic data where a feature is described by its mass to charge ratio and retention time. Figure 3(A), representing the CVF metabolome’s heatmap prior to treatment, shows a complex mosaic of alternating positive and negative correlations, with few strong correlations. This suggests numerous overlapping clusters of features with an intertwined correlation structure. In contrast, Fig. 3(B), which represents the CVF metabolome immediately post-pessary removal, exhibits larger, well-defined blocks of strong positive correlations along the diagonal which are also separated by clear regions of negative association. Thus, post-pessary removal, there is a clearer indication of structured correlation, which supports Fig. 2.

Table 4 robustly characterises the statistical significance of detected features where only features with *P* < 0.005, FDR < 0.05 and iFit Confidence > 80% were reported. For completeness, the full set of results underlying Table 4 is provided in Supplementary Table S1. A total of 32 features were reported, of which one was uniquely identified at level 1, one at level 2, 24 at level 3 and 6 at level 4. Progesterone was identified as a level 1 feature and was significantly higher in the CIDR-treated group compared to both the sponge-treated and control groups (P(Control vs. CIDR-treated, sponge- vs. CIDR-treated) < 5.00 × 10^− 13^), with no difference between the sponge-treated and control group (*P* > 0.05). This feature was also identified on level 2 as Androstan-3α,17beta-diol; 17α- Hydroxypregnenolone; Tetrahydrocorticosterone; 21-Hydroxypregnenolone; 7α-Hydroxypregnenolone; 5α-Dihydrodeoxycorticosterone and 5α-Pregnan-17α-ol-3,20-dione. Linolenic acid was identified at level 2 (*P* < 0.0005), and its concentration was higher in ewes treated with sponges compared to those in the control group. The twenty-four features identified at level 3 had chemical formulas ranging in iFit Confidence from 10.72% to 100%. The range of these chemical formulas indicates small molecules (ca. 280 g/mol) to large peptides and complex drugs (> 800 g/mol). Of the six features identified at level 4, only one (797.2303_16.926) was higher in the sponge-treated group (*P* < 0.0005).

**Table 4:** Summary of the overall and treatment specific significance as well as identification level of detected features in cervicovaginal secretions of Dohne Merino ewes immediately post-CIDR, sponge or control treatment (full results in Supplementary Table S1)

m/z^1^	rt^2^	*P* ^3^	FDR^4^	diff^5^ (Control-CIDR)^7^	Sig.^6^ (Control-CIDR)^7^	diff^5^ (Sponge-CIDR)^8^	Sig.^6^ (Sponge-CIDR)^8^	diff^5^ (Sponge-Control)^9^	Sig.^6^ (Sponge-Control)^9^	ID Level^10^	Name	KEGG ID	Adduct	Chemical formula	iFit^11^	Mass Diff^12^
315.2303	9.128	7.72 × 10^− 48^	2.4 × 10^− 45^	-9.640	1.31 × 10^− 14^	-9.970	1.31 × 10^− 14^	-0.327	0.750	1	Progesterone	C00410	[M + Na]^+^	C_21_H_30_O_2_		
										2	Androstan-3α,17beta-diol	C03852		C_19_H_32_O_2_		0.000854
											17α-Hydroxypregnenolone	C05138	[M-CO + H]^+^	C_21_H_32_O_3_		0.001446
											Tetrahydrocorticosterone	C05476	[M-H_4_O_2_+H]^+^	C_21_H_34_O_4_		
											21-Hydroxypregnenolone	C05485	[M-H_2_O + H]^+^	C_21_H_32_O_3_		
											7α-Hydroxypregnenolone	C18038		C_21_H_32_O_3_		
											5α-Dihydrodeoxycorticosterone	C18040		C_21_H_32_O_3_		
											5α-Pregnan-17α-ol-3,20-dione	C22831		C_21_H_32_O_3_		
451.2132	7.296	2.01 × 10^− 36^	1.63 × 10^− 34^	0.437	0.663	8.510	1.31 × 10^− 14^	8.070	1.31 × 10^− 14^	3				C_22_H_32_F_4_O_5_	98.29	
441.1852	7.297	2.53 × 10^− 28^	8.23 × 10^− 27^	0.391	0.756	7.370	1.31 × 10^− 14^	6.98	1.31 × 10^− 14^	3				C_21_H_29_CIF_2_N_4_O_2_	85.12	
														C_23_H_32_CIFO_5_	11.84	
355.2617	7.271	2.19 × 10^− 12^	5.48 × 10^− 11^	-3.130	4.55 × 10^− 4^	3.470	8.92 × 10^− 5^	6.600	8.47 × 10^− 13^	3				C_24_H_34_O_2_	92.91	
302.3024	8.164	6.47 × 10^− 5^	0.001	-0.261	0.474	-0.986	6.59 × 10^− 5^	-0.725	0.004	3				C_18_H_39_NO_2_	100.00	
522.3550	11.713	1.08 × 10^− 4^	0.002	-0.098	0.978	-1.950	3.97 × 10^− 4^	-1.850	8.11 × 10^− 4^	3				C_25_H_48_FN_3_O_7_	80.91	
														C_26_H_52_NO_7_P	16.96	
301.2147	5.575	2.36 × 10^− 4^	0.004	-1.000	0.144	1.230	0.055	2.230	1.38 × 10^− 4^	2	Linolenic acid	C06426	[M + Na]+	C_18_H_30_O_2_		0.000784

^1^Mass-to-charge ratio (m/z)

^2^Retention time (rt)

^3^ANOVA P-value (P)

^4^False Discovery Rate (FDR)

^5^Tukey’s HSD (diff) per group comparison

^6^Significance (Sig.) per group comparison

^7^Control group compared to CIDR-treated group (Control-CIDR)

^8^Sponge-treated compared to CIDR-treated group (Sponge-CIDR)

^9^Sponge-treated compared to control group (Sponge-Control)

^10^Level of identification (ID Level)

^11^iFit Confidence (iFit) (%)

^12^Mass difference standard deviation (Mass Diff) (m/z)

## Discussion

The current study used metabolomic LC-HRMS analysis to profile the cervicovaginal metabolome of Dohne Merino ewes, and pioneer a novel understanding of both homeostatic and dysbiotic CVF metabolomes triggered by the administration of intravaginal pessaries for estrus synchronisation in small stock. The performance of livestock may be affected by various factors, both innate and external to the animal, that present challenges to their homeostatic maintenance of various body systems (Tsiligianni et al. 2001). Homeostasis and the mechanisms used to attain and maintain it depend on an innumerable number of metabolites that correlate with its physiological state. In the case of estrus synchronisation, the results from this study indicate that ewes treated with intravaginal pessaries establish an alternate cervicovaginal metabolome depending on the method of synchronisation, namely, CIDR- or sponge-based treatment compared to those synchronised only with injected hormones. The magnitude of the effect of the treatment, in the case of sponge treatment, increased by 25% prior to pessary treatment compared to immediately post-pessary removal (ΔR² (CIDR-treated vs. Sponge-treated, Control vs. Sponge-treated) ca. 0.252).

The increased abundance of progesterone in the CIDR-treated group immediately post-pessary removal is likely due to extruded progesterone from the CIDR itself. This notion contradicts literature that found that sponge-pessaries release more progesterone at pessary removal due to the squeezing effect as it is pulled from the female’s vaginal canal through the vulva (Bartlewski et al. 2015) while supporting literature that found increased progesterone in CIDR treated vs. sponge treated groups (Hamisi et al. 2023). This finding is contextually relevant to livestock production systems where a CIDR is reused for more than one estrus synchronisation protocol, as found in various studies (Pinna et al. 2012; Bragança et al. 2017).

The corpus luteum is the primary source of endogenous progesterone in female livestock (Okon et al. 2016; Bihon and Assefa 2021). Because the length of progestagen synchronisation protocols in ewes may span over more than one phase of estrous, it is possible that the detected progesterone was not due to the CIDR but rather a product of the ewes’ own metabolism. In interrogating this claim, a primary consideration is the interaction of the CIDR or sponge with the ewe’s ovarian structures at the commencement of treatment. If an ewe was in estrus, metestrus or diestrus when treatment commenced, the developing or developed corpus luteum will actively secrete progesterone until treatment ceases (Amiridis and Cseh 2012; Santos et al. 2017; Dogan et al. 2018). However, these devices do not enhance the growth of or prolong the lifespan of the corpus luteum by increasing luteal blood flow, enhancing steroidogenic capacity or delaying luteolysis (Leyva et al. 1998; Pugliesi et al. 2014; Cosentino et al. 2023). If an ewe was in proestrus when treatment commenced, her corpus luteum had begun regression to allow for the development of a dominant Graafian follicle and the CIDR or sponge would not inhibit this luteolysis (Kojima et al. 1992; Garcia-Muñoz et al. 2017). Thus, this study asserts that the significant difference in progesterone concentration in the CIDR-treated group was not of an endogenous source because exogenous progesterone released from the CIDR or sponge dominates circulating progesterone during treatment and these devices do not support the corpus luteum. This argument is further supported by the control group having lower progesterone than both the treatment groups.

Linolenic acid was found to be significantly higher in the sponge-treated group compared to the CIDR-treated and control group. It is an omega-3 polyunsaturated fatty acid (PUFA) that must be obtained from diet and cannot be synthesised by the body (Calder 2020; Toral et al. 2024). As a dietary component, it can be mobilised in its liposomal form to act rapidly against bacterial membranes and play an important anti-inflammatory role during infection (Jung et al. 2015; Toral et al. 2024; Contreras-Solís et al. 2025). In interpreting how PUFA shifts may influence immune outcomes, literature has shown that the ewes’ vaginal microbiome itself varies with reproductive factors and interacts with host immunity (Esposito et al. 2014; Reinoso-Peláez et al. 2025). As the mucosal immune system in genital tracts is an active barrier with cytokines, antimicrobial peptides and immune cells that can respond to microbial and biochemical signals (Dadarwal et al. 2017), this paper asserts that the immune mechanisms responsible for maintaining the ewes’ cervicovaginal environment were triggered by the sponge-treatment.

Further, linolenic acid’s significance holds unique animal welfare implications for the use of sponges as this PUFA was found to have exceptional antibiofilm activity when compared to eicosapentaenoic acid and docosahexaenoic acid (Wei et al. 2023). As noted in other literature, the intra-vaginal sponge triggers vaginal discharge that is hemorrhagic, putrid and/ dysbiotic (Scudamore 1988; Shallali et al. 2001; Manes et al. 2014). Thus, because the sponge is made of polyurethane which has been proven to have a higher propensity for biofilm formation even compared to silicon of the CIDR (Ribeiro et al. 2003), the increase of linolenic acid in the sponge-treated group may be a treatment specific response.

Lastly, the features that were identified on level 3 or 4 were speculatively phosphorylated metabolites, thiophosphate-modified nucleotides, fluorinated small molecules and lipid/phospholipids or peptides that are arginine rich with a vast range of physiological roles. Despite classification of metabolites based on carbon-number being a valid method of separating lipids, aromatics, peptides etc. (Reemtsma 2010), annotations are tentative and further identification is necessary. Increased phosphorylated metabolites or those that contain phosphonate groups in the sponge-treated group, for example, C_20_H_28_FN_2_O_4_P_2_ (461.1518_3.094) at 21.20% iFit Confidence, may reflect altered epithelial metabolism due to increased ATP or ADP after progesterone exposure because progesterone alters local epithelial secretion and immune signalling (Dias 2022; Kolatorova et al. 2022). This is similar for fluorinated small molecules such as C_22_H_32_F_4_O_5_ (451.2132_7.296) (Maxwell et al. 2024), determined at 98.29% iFit Confidence, which was highest in the sponge-treated group. Similarly, peptides, lipids and phospholipids such as C_38_H_52_N_16_O (813.4426_7.299) at 12.91% iFit Confidence, alter membrane function as bioactive lipids have arginine and lysine rich peptides as a common part of the immune system in mucosal surfaces (Shan et al. 2021; Zhang et al. 2025). This latter finding holds promise for further research into immune-specific characterisation of the effects of intra-vaginal pessary treatments as these metabolites were highest in the sponge-treated group. Surprisingly, thiophosphate-modified nucleotides are not endogenous metabolites. Rather, they are synthetic backbone modifications used to stabilise biological fluids and may stimulate local immunity and alter cytokine profiles (Kurreck 2002; Pollak et al. 2022). This finding is crucial as sponge intravaginal pessaries are cited as triggering adverse immune responses in the vaginal environment (Bragança et al. 2017; Dias 2022). Thus, the detection of C_14_H_26_FN_6_O_3_PS (409.1564_3.970) at 10.48% iFit Confidence, a potential thiophosphate nucleotide as it bears sulphur in its phosphate group, supports this notion.

Identified and annotated features have thus unveiled metabolites across a range of physiological roles with even broader practical implications for the reproductive management of livestock. The sponge triggers a change in the CVF metabolome of ewes that is significantly different from that of ewes treated with a CIDR or without a pessary. For animal welfare, this change supports the use of CIDRs over sponges due a lower immune response as correlated to linolenic acid concentrations and, speculatively, to metabolites identified at level 3 and 4. For efficiency of progesterone delivery, this change also supports the use of CIDRs over sponges due to their increased progesterone concentrations at intra-vaginal pessary removal.

## Conclusion

For an organism to maintain its internal environment, it must use a plethora of metabolites from different chemical classes, each with unique and sometimes overlapping roles in various physiological cascades. As such, a change in the metabolomic composition of a microenvironment necessarily reflects a change in underlying genetic transcription aimed at the ultimate manifestation of a phenotype that is nearest to homeostasis. This study indicates that, firstly, the CIDR and sponge alter the cervicovaginal metabolome of Dohne Merino ewes when compared to prior to treatment as shown by an increase in number of features that were found to be significantly different between treatment groups compared to the lack of significant features prior to treatment. Secondly, this study presents novel evidence of the dysbiosis caused by intra-vaginal pessaries, and sponge pessaries in particular, as shown by the identification of metabolites associated with immune response. This study represents a unique metabolomic approach to the assessment of routine livestock reproductive protocols and capitalises on metabolomics’ capacity to elucidate the correlation between genetics and manifestation of phenotypes. This study recommends increased industry and scientific focus on level 1 and 2 identification of metabolites in the various body systems of livestock as the foundation of accurate reporting of the vast roles that these metabolites play and the factors that affect their increase, decrease or maintenance. This identification will play a continued role in enhancing the efficiency of production practices and upholding animal welfare.

## Supplementary Information

Below is the link to the electronic supplementary material.

Supplementary Material 1

## Data Availability

The datasets generated during and/or analysed during the current study are not publicly available as they are temporarily confidential until completion of the thesis marking process. Access may be granted by the corresponding author upon reasonable request.
