# Supplementary material for: Differential metabolomic effects of progestagen-based estrus synchronisation in South African Dohne Merino ewes
Source: Trop Anim Health Prod. 2026 Jul 10;58(6):407. doi: 10.1007/s11250-026-05198-0 (PMC13354613; doi:10.1007/s11250-026-05198-0)
Supplement: Supplementary file 1 — Supplementary Material 1 [file 11250_2026_5198_MOESM1_ESM.docx]

**Differential metabolomic effects of progestagen-based estrus synchronisation in South African Dohne Merino ewes**

R. Shingange^1*^, C. Visser^1^, M. Wooding^2^, E. C. Webb^1^

^1^Department of Animal Science, University of Pretoria, Pretoria, South Africa

^2^Department of Chemistry, University of Pretoria, Pretoria, South Africa

*Corresponding author: *rimbilana.shingange@up.ac.za*

**Supplementary material**

1. **Instrument method**

Compound separation and detection were performed using a Waters^®^ Synapt G2 [high definition mass spectrometry](http://www.waters.com/waters/en_US/MALDI-SYNAPT-G2-Si-High-Definition-Mass-Spectrometry/nav.htm?cid=134740682) (HDMS) system (Waters Inc., Milford, Massachusetts, USA). The system comprises of a Waters Acquity Ultra Performance Liquid Chromatography (UPLC^®^) system hyphenated to a quadrupole-time-of-flight (QTOF) instrument. The system was operated with MassLynx™ (version 4.1) software (Waters Inc., Milford, Massachusetts, USA) for data acquisition and processing. The source conditions were as follows: the capillary voltage for ESI was 2.6 kV and 2.0 kV for positive and negative mode ionisation. The source temperature was set at 120 ºC, the sampling cone voltage at 25 V, extraction cone voltage at 4.0 V and cone gas (nitrogen) flow at 10.0 L/Hr. The desolvation temperature was set at 350 ºC with a gas (nitrogen) flow of 600.0 L/Hr.

Quantitative data-independent acquisition (DIA) was done using two simultaneous acquisition functions with low and high collision energy (MS^E^ approach) with a QTOF instrument. Fragmentation was performed using high-energy collision-induced dissociation (CID). The fragmentation energy was set at 2 V and 3 V for the trap and collision energy, respectively. The ramping was set from 3 to 4 V and 20 to 40 V for the trap and transfer collision energy, respectively. Mass spectral scans were collected every 0.3 seconds. The raw data was collected in the form of a continuous profile. Mass to charge ratios (*m/z*) between 50 and 1 200 Da were recorded.

Separation was completed using a reverse phase step gradient elution scheme from 97% H2O (0.1% formic acid) to 100% acetonitrile (0.1% formic acid). The gradient started with an isocratic flow (hold 0.1 min) followed by a linear increase to 100% ACN; subsequently the column was washed for 1 min followed by conditioning and re-establishing of initial conditions to allow for equilibration before the start of the next run for the complete elution scheme. The column temperature was kept constant at 40 ºC and the flow rate was set at 0.4 mL/min for the entire run giving a total run time of 20 min. Injection volumes were set at 5 µL. A Kinetex® 1.7 µm EVO C18 100 Å (2.1 mm ID x 100 mm length) column was used. The positive and negative ion mass spectra were collected in separate chromatographic runs (employing the same separation conditions).

1. **Supplementary Table S1:**

**Full dataset corresponding to the summary statistics in Table 4 (The overall and treatment specific significance as well as identification level of detected features in cervicovaginal secretions of Dohne-Merino ewes immediately post-CIDR, sponge or control treatment)**

| m/z^1^ | rt^2^ | P^3^ | FDR^4^ | diff^5^  (Control-CIDR)^7^ | Sig.^6^  (Control-CIDR)^7^ | diff^5^  (Sponge-CIDR)^8^ | Sig.^6^  (Sponge-CIDR)^8^ | diff^5^  (Sponge-Control)^9^ | Sig.^6^ (Sponge-Control)^9^ | ID Level^10^ | Name | KEGG ID | Adduct | Chemical  formula | iFit^11^ | Mass Diff^12^ |  |  |  |
| --- | --- | --- | --- | --- | --- | --- | --- | --- | --- | --- | --- | --- | --- | --- | --- | --- | --- | --- | --- |
| 315.2303 | 9.128 | 7.72x10^-48^ | 2.4x10^-45^ | -9.640 | 1.31x10^-14^ | -9.970 | 1.31x10^-14^ | -0.327 | 0.750 | 1 | Progesterone | C00410 | [M+Na]^+^ | C_21_H_30_O_2_ |  |  |  |  |  |
|  |  |  |  |  |  |  |  |  |  | 2 | Androstan-3α,17beta-diol | C03852 |  | C_19_H_32_O_2_ |  | 0.000854 |  |  |  |
|  |  |  |  |  |  |  |  |  |  |  | 17α-Hydroxypregnenolone | C05138 | [M-CO+H]^+^ | C_21_H_32_O_3_ |  | 0.001446 |  |  |  |
|  |  |  |  |  |  |  |  |  |  |  | Tetrahydrocorticosterone | C05476 | [M-H_4_O_2_+H]^+^ | C_21_H_34_O_4_ |  |  |  |  |  |
|  |  |  |  |  |  |  |  |  |  |  | 21-Hydroxypregnenolone | C05485 | [M-H_2_O+H]^+^ | C_21_H_32_O_3_ |  |  |  |  |  |
|  |  |  |  |  |  |  |  |  |  |  | 7α-Hydroxypregnenolone | C18038 |  | C_21_H_32_O_3_ |  |  |  |  |  |
|  |  |  |  |  |  |  |  |  |  |  | 5α-Dihydrodeoxycorticosterone | C18040 |  | C_21_H_32_O_3_ |  |  |  |  |  |
|  |  |  |  |  |  |  |  |  |  |  | 5α-Pregnan-17α-ol-3,20-dione | C22831 |  | C_21_H_32_O_3_ |  |  |  |  |  |
| 407.2196 | 7.301 | 1.48x10^-47^ | 2.40x10^-45^ | 0.200 | 0.907 | 10.400 | 1.31x10^-14^ | 10.200 | 1.31x10^-14^ | 3 |  |  |  | C_20_H_31_N_4_O_3_P | 64.31 |  |  |  |  |
| 387.1745 | 3.971 | 1.54x10^-41^ | 1.67x10^-39^ | -0.002 | 1.000 | 8.680 | 1.31x10^-14^ | 8.690 | 1.31x10^-14^ | 3 |  |  |  | C_16_H_29_F_2_O_6_P | 79.50 |  |  |  |  |
| 451.2132 | 7.296 | 2.01x10^-36^ | 1.63x10^-34^ | 0.437 | 0.663 | 8.510 | 1.31x10^-14^ | 8.070 | 1.31x10^-14^ | 3 |  |  |  | C_22_H_32_F_4_O_5_ | 98.29 |  |  |  |  |
| 409.1564 | 3.970 | 5.45x10^-36^ | 3.54x10^-34^ | 0.0632 | 0.989 | 7.180 | 1.31x10^-14^ | 7.120 | 1.31x10^-14^ | 3 |  |  |  | C_16_H_29_F_4_O_3_PS | 11.55 |  |  |  |  |
|  |  |  |  |  |  |  |  |  |  |  |  |  |  | C_15_H_25_F_5_N_2_O_3_S | 11.37 |  |  |  |  |
|  |  |  |  |  |  |  |  |  |  |  |  |  |  | C_14_H_26_FN_6_O_3_PS | 10.48 |  |  |  |  |
|  |  |  |  |  |  |  |  |  |  |  |  |  |  | C_15_H30N_4_O_3_P_2_S | 10.34 |  |  |  |  |
| 347.1972 | 7.299 | 1.86x10^-35^ | 1.01x10^-33^ | 0.0902 | 0.976 | 6.920 | 1.31x10^-14^ | 6.830 | 1.31x10^-14^ | 3 |  |  |  | C_21_H_27_FO_3_ | 67.50 |  |  |  |  |
|  |  |  |  |  |  |  |  |  |  |  |  |  |  | C_18_H_28_F_2_O_4_ | 13.81 |  |  |  |  |
| 461.1518 | 3.094 | 2.49x10^-33^ | 1.15x10^-31^ | 0.397 | 0.781 | 9.140 | 1.31x10^-14^ | 8.740 | 1.31x10^-14^ | 3 |  |  |  | C_20_H_28_FN_2_O_4_P_2_ | 21.20 |  |  |  |  |
|  |  |  |  |  |  |  |  |  |  |  |  |  |  | C_21_H_32_FO_4_P_3_ | 21.10 |  |  |  |  |
|  |  |  |  |  |  |  |  |  |  |  |  |  |  | C_19_H_24_F_3_N_4_O_4_P | 13.31 |  |  |  |  |
| 813.4426 | 7.299 | 3.38x10^-31^ | 1.37x10^-29^ | 0.403 | 0.741 | 7.970 | 1.31x10^-14^ | 7.570 | 1.31x10^-14^ | 3 |  |  |  | C_43_H_74_FO_3_PS | 13.35 |  |  |  |  |
|  |  |  |  |  |  |  |  |  |  |  |  |  |  | C_38_H_52_N_16_O | 12.91 |  |  |  |  |
|  |  |  |  |  |  |  |  |  |  |  |  |  |  | C_40_H_55_F_3_N_10_O_5_ | 12.78 |  |  |  |  |
|  |  |  |  |  |  |  |  |  |  |  |  |  |  | C_42_H_58_F_6_N_4_O_5_ | 11.89 |  |  |  |  |
|  |  |  |  |  |  |  |  |  |  |  |  |  |  | C_42_H_70_F_2_N_2_O_3_P_4_ | 10.49 |  |  |  |  |
| 297.1547 | 8.745 | 7.97x10^-29^ | 2.88x10^-27^ | -0.482 | 0.707 | 7.830 | 1.35x10^-14^ | 8.310 | 1.31x10^-14^ | 3 |  |  |  | C_14_H_23_FN_4_S | 67.94 |  |  |  |  |
|  |  |  |  |  |  |  |  |  |  |  |  |  |  | C_15_H_27_N_2_PS | 30.81 |  |  |  |  |
| 441.1852 | 7.297 | 2.53x10^-28^ | 8.23x10^-27^ | 0.391 | 0.756 | 7.370 | 1.31x10^-14^ | 6.98 | 1.31x10^-14^ | 3 |  |  |  | C_21_H_29_CIF_2_N_4_O_2_ | 85.12 |  |  |  |  |
|  |  |  |  |  |  |  |  |  |  |  |  |  |  | C_23_H_32_CIFO_5_ | 11.84 |  |  |  |  |
| 453.3421 | 4.176 | 8.68x10^-17^ | 2.56x10^-15^ | -2.600 | 4.6x10^-14^ | -2.320 | 8.65x10^-13^ | 0.284 | 0.571 | 3 |  |  |  | C_18_H_45_N_8_O_3_P | 59.17 |  |  |  |  |
|  |  |  |  |  |  |  |  |  |  |  |  |  |  | C_21_H_45_FN_4_OS | 20.39 |  |  |  |  |
| 475.3250 | 4.180 | 1.52x10^-12^ | 4.11x10^-11^ | -531.000 | 2.15x10^-12^ | -3.820 | 1.63x10^-7^ | 1.480 | 0.070 | 3 |  |  |  | C_22_H_38_N_10_O_2_ | 31.78 |  |  |  |  |
|  |  |  |  |  |  |  |  |  |  |  |  |  |  | C_24_H_41_F_3_N_4_O_2_ | 21.12 |  |  |  |  |
|  |  |  |  |  |  |  |  |  |  |  |  |  |  | C_25_H_4_F_2_N_2_O_2_P | 14.08 |  |  |  |  |
| 355.2617 | 7.271 | 2.19x10^-12^ | 5.48x10^-11^ | -3.130 | 4.55 x 10^-4^ | 3.470 | 8.92x10^-5^ | 6.600 | 8.47x10^-13^ | 3 |  |  |  | C_24_H_34_O_2_ | 92.91 |  |  |  |  |
| 463.2887 | 13.464 | 2.06x10^-8^ | 4.77x10^-7^ | -4.960 | 1.87x10^-7^ | -4.570 | 1.46x10^-6^ | 0.390 | 0.890 | 3 |  |  |  | C_28_H_40_F_2_O_3_ | 11.69 |  |  |  |  |
|  |  |  |  |  |  |  |  |  |  |  |  |  |  | C_23_H_38_N_6_O_4_ | 11.20 |  |  |  |  |
|  |  |  |  |  |  |  |  |  |  |  |  |  |  | C_25_H_41_F_3_O_4_ | 10.74 |  |  |  |  |
| 522.3551 | 11.292 | 2.14x10^-6^ | 4.64x10^-5^ | -1.640 | 0.002 | -2.520 | 1.45x10^-6^ | -0.884 | 0.152 | 3 |  |  |  | C_25_H_48_FN_3_O_7_ | 46.94 |  |  |  |  |
|  |  |  |  |  |  |  |  |  |  |  |  |  |  | C_26_H_52_NO_7_P | 17.72 |  |  |  |  |
| 496.3388 | 11.263 | 2.51x10^-6^ | 5.11x10^-5^ | 0.455 | 0.654 | -2.140 | 1.87x10^-4^ | -2.600 | 5.35x10^-6^ | 3 |  |  |  | C_32_H_43_F_2_NO | 52.84 |  |  |  |  |
|  |  |  |  |  |  |  |  |  |  |  |  |  |  | C_27_H_41_N_7_O_2_ | 11.23 |  |  |  |  |
|  |  |  |  |  |  |  |  |  |  |  |  |  |  | C_29_H_44_F_3_NO_2_ | 10.03 |  |  |  |  |
| 302.3024 | 8.164 | 6.47x10^-5^ | 0.001 | -0.261 | 0.474 | -0.986 | 6.59x10^-5^ | -0.725 | 0.004 | 3 |  |  |  | C_18_H_39_NO_2_ | 100.00 |  |  |  |  |
| 522.3550 | 11.713 | 1.08x10^-4^ | 0.002 | -0.098 | 0.978 | -1.950 | 3.97x10^-4^ | -1.850 | 8.11x10^-4^ | 3 |  |  |  | C_25_H_48_FN_3_O_7_ | 80.91 |  |  |  |  |
|  |  |  |  |  |  |  |  |  |  |  |  |  |  | C_26_H_52_NO_7_P | 16.96 |  |  |  |  |
| 480.3053 | 9.842 | 1.45x10^-4^ | 0.002 | -3.200 | 9.6x10^-5^ | -1.190 | 0.244 | 2.000 | 0.021 | 3 |  |  |  | C_34_H_38_FN | 39.16 |  |  |  |  |
|  |  |  |  |  |  |  |  |  |  |  |  |  |  | C_31_H_39_F_2_NO | 10.84 |  |  |  |  |
| 797.2303 | 16.926 | 2.16x10^-4^ | 0.004 | 2.040 | 0.0248 | 3.250 | 1.46x10^-4^ | 1.210 | 0.264 | 4 |  |  |  |  |  |  |  |  |  |
| 301.2147 | 5.575 | 2.36x10^-4^ | 0.004 | -1.000 | 0.144 | 1.230 | 0.055 | 2.230 | 1.38x10^-4^ | 2 | Linolenic acid | C06426 | [M+Na]+ | C_18_H_30_O_2_ |  | 0.000784 |  |  |  |
| 631.3549 | 9.059 | 0.001 | 0.014 | -0.247 | 0.909 | -2.080 | 0.0018 | -1.830 | 0.007 | 4 |  |  |  |  |  |  |  |  |  |
| 496.3382 | 10.835 | 0.001 | 0.015 | -1.740 | 0.003 | -1.730 | 0.004 | 0.012 | 1.000 | 3 |  |  |  | C_32_H_43_F_2_NO | 52.84 |  |  |  |  |
|  |  |  |  |  |  |  |  |  |  |  |  |  |  | C_27_H_41_N_7_O_2_ | 11.23 |  |  |  |  |
|  |  |  |  |  |  |  |  |  |  |  |  |  |  | C_29_H_44_F_3_NO_2_ | 10.03 |  |  |  |  |
| 1011.1280 | 13.117 | 0.002 | 0.022 | 0.040 | 0.996 | -1.43 | 0.006 | -1.460 | 0.004 | 4 |  |  |  |  |  |  |  |  |  |
| 373.2836 | 6.26 | 0.002 | 0.030 | -0.846 | 0.069 | 0.485 | 0.408 | 1.330 | 0.002 | 3 |  |  |  | C_23_H_36_N_2_O_2_ | 48.27 |  |  |  |  |
|  |  |  |  |  |  |  |  |  |  |  |  |  |  | C_22_H_39_F_2_P | 23.97 |  |  |  |  |
|  |  |  |  |  |  |  |  |  |  |  |  |  |  | C_20_H_37_FN_2_O_3_ | 15.79 |  |  |  |  |
| 650.8876 | 9.056 | 0.002 | 0.030 | -0.406 | 0.814 | -2.22 | 0.003 | -1.820 | 0.0197 | 4 |  |  |  |  |  |  |  |  |  |
| 409.1823 | 3.006 | 0.003 | 0.037 | -0.424 | 0.405 | 0.708 | 0.084 | 1.130 | 0.002 | 3 |  |  |  | C_14_H_32_F_3_N_4_PS_2_ | 15.49 |  |  |  |  |
|  |  |  |  |  |  |  |  |  |  |  |  |  |  | C_12_H_29_N_10_PS_2_ | 12.02 |  |  |  |  |
|  |  |  |  |  |  |  |  |  |  |  |  |  |  | C_15_H_29_FN_6_O_2_S_2_ | 10.77 |  |  |  |  |
|  |  |  |  |  |  |  |  |  |  |  |  |  |  | C_13_H_28_F_4_N_6_S_2_ | 10.67 |  |  |  |  |
| 586.4522 | 10.317 | 0.003 | 0.037 | 1.530 | 0.015 | -0.167 | 0.948 | -1.690 | 0.006 | 4 |  |  |  |  |  |  |  |  |  |
| 684.4577 | 9.078 | 0.003 | 0.037 | 0.944 | 0.429 | -1.650 | 0.0805 | -2.590 | 0.003 | 3 |  |  |  | C_42_H_66_CINO_2_S | 11.26 |  |  |  |  |
|  |  |  |  |  |  |  |  |  |  |  |  |  |  | C_45_H_62_CINO_2_ | 10.79 |  |  |  |  |
| 727.9962 | 16.935 | 0.003 | 0.037 | 0.636 | 0.403 | -1.050 | 0.090 | -1.680 | 0.003 | 4 |  |  |  |  |  |  |  |  |  |
| 520.3363 | 9.979 | 0.004 | 0.037 | -0.574 | 0.366 | -1.440 | 0.002 | -0.869 | 0.103 | 3 |  |  |  | C_29_H_41_N_7_O_2_ | 36.87 |  |  |  |  |
|  |  |  |  |  |  |  |  |  |  |  |  |  |  | C_34_H_43_F_2_NO | 25.25 |  |  |  |  |
|  |  |  |  |  |  |  |  |  |  |  |  |  |  | C_28_H_45_N_3_O_6_ | 21.28 |  |  |  |  |
| 712.0036 | 13.357 | 0.004 | 0.042 | 0.174 | 0.033 | -0.046 | 0.782 | -0.220 | 0.005 | 4 |  |  |  |  |  |  |  |  |  |
| ^1^Mass-to-charge ratio (m/z)  ^2^Retention time (rt)  ^3^ANOVA P-value (P)  ^4^False Discovery Rate (FDR)  ^5^Tukey’s HSD (diff) per group comparison  ^6^Significance (Sig.) per group comparison  ^7^Control group compared to CIDR-treated group (Control-CIDR)  ^8^Sponge-treated compared to CIDR-treated group (Sponge-CIDR)  ^9^Sponge-treated compared to control group (Sponge-Control)  ^10^Level of identification (ID Level)  ^11^iFit Confidence (iFit) (%)  ^12^Mass difference (Mass Diff) (m/z) | | | | | | | | | | | | | | | | | |  |  |
